# Clinical pathways for inborn errors of metabolism: warranted and feasible

**DOI:** 10.1186/1750-1172-8-37

**Published:** 2013-02-25

**Authors:** Serwet Demirdas, Imke N van Kessel, Marjolein J Korndewal, Carla EM Hollak, Hanka Meutgeert, Anja Klaren, Margreet van Rijn, Francjan J van Spronsen, Annet M Bosch

**Affiliations:** 1 Department of Pediatrics, Emma Children’s Hospital, Academic Medical Center, University of Amsterdam, Amsterdam, The Netherlands; 2 Department of Internal Medicine, Division of Endocrinology and Metabolism, Academic Medical Center, University of Amsterdam, Amsterdam, The Netherlands; 3 The Dutch Society for Adults and Children with an Inborn Error of Metabolism (VKS), Zwolle, The Netherlands; 4 Division of Metabolic Diseases, Beatrix Children’s Hospital, University Medical Center Groningen, University of Groningen, Groningen, The Netherlands

**Keywords:** Phenylketonuria, PKU, Clinical pathway

## Abstract

Inborn errors of metabolism (IEMs) are known for their low prevalence and multidisciplinary care mostly founded on expert opinion. Clinical pathways are multidisciplinary tools to organise care which provide a clear route to the best care and improve communication. In 2010 the Dutch Society for Children and Adults with an Inborn Error of Metabolism (VKS) initiated development of clinical pathways for inborn errors of metabolism. In this letter to the editor we describe why it is warranted to develop clinical pathways for IEMs and shortly discuss the process of development for these pathways in the Netherlands.

## Introduction

Inborn errors of metabolism (IEMs) are known for their low prevalence and chronic need of medical care. Care provided is multidisciplinary and often based on expert opinion. In recent years, excellent guidelines on metabolic disorders have been developed and published [1-5]. In 2010 the Dutch Society for Children and Adults with an Inborn Error of Metabolism (VKS) initiated development of clinical pathways for 20 IEMs, with separate versions for professionals and patients. This letter discusses why clinical pathways for IEMs are warranted and feasible.

### Background

Clinical pathways are a tool for multidisciplinary decision making and organization of care processes for well defined groups of patients [6]. Often they are based on guidelines [7-9]. Pathways optimize clinical outcomes whilst maximizing clinical efficiency [10]. For example, they describe which actions should be taken, when, and by whom [11]. It has been demonstrated that use of pathways decreases duration of inpatient care, increases interdisciplinary communication, enhances patient knowledge and self awareness, leads to significant better coordination of care and reduces costs [7,9,12-14].

Clinical pathways can be valuable for patients with IEMs. Firstly, low prevalence of IEMs leads to limited knowledge about best practice. In the absence of robust evidence, expert opinion and outcomes of clinical studies can support the establishment of a clinical pathway [12]. When frequently updated, it presents a reference to latest state of art in care [15,16] and provides guidance for further research. Secondly, a multidisciplinary approach is of great importance. The complexity of multidisciplinary care may lead to delay of care, overuse of diagnostics or therapy and miscommunication between caregivers [7]. Multidisciplinary cooperation using a clinical pathway will improve communication and provide a clear route to best care, based on consensus. Thirdly, clinical pathways may improve care for patients when used in local hospitals, while the physicians in academic referral centers can serve as consultants. Finally, clinical pathways become more important as transition to adult care increases [17], leading to more active participation of patients in their treatment.

### The design of clinical pathways for inborn errors of metabolism

#### Design and consensus

The initiative for development of clinical pathways was taken by the patient society (VKS). Dutch expert pediatricians, internists and dieticians for each specific disorder in cooperation with the VKS created the pathways. The final version was discussed in a national consensus meeting. Separate versions were made for professionals and for patients, presenting the Dutch consensus. All advice is substantiated by a level of evidence [18], according to the scoring system of the Dutch Institute for Healthcare Improvement CBO. Level 1: 1 systematic review or 2 independent high quality randomized controlled trials (RCTs); level 2: 2 independent moderate RCTs or comparative trials; Level 3: 1 RCT, comparative or non-comparative trial; Level 4: expert opinion [19].

#### Clinical pathway for professionals

The first section of the version for professionals comprises a general introduction and concise strategy for diagnostics and treatment. Second and third sections contain more specific guidance for treatment and follow up in childhood and adulthood.

The pathways include responsibilities for each professional, advised frequency for outpatient visits and laboratory studies, and recommendations on follow up of known complications of the disorder. In the pediatric pathway one chapter is dedicated to transition from pediatric to adult care.

In the pathways all advice is substantiated by a level of evidence. Evidence levels 3 and 4 were common. Level 1 was rarely available and mostly resulted from trials evaluating a novel pharmaceutical agent. Most advice was therefore founded on expert opinion and trials of moderate quality.

#### Clinical pathway for patients

The first section of the patient version contains general information on the disorder and its treatment. The second and third sections address treatment and follow up in childhood and adulthood. The purpose of the pathway for patients is to provide insight into current consensus of best practice and an overview of all professionals involved. It provides clarity on responsibilities, including that of the patient/parents who take a prominent place in the treatment team.

For active patient participation, patients must be provided evidence based information in an appropriate and comprehensible form [20]. The fact that the patient versions are based on the professional pathway ensures that they are in accordance with available evidence, and comprehensibility is secured by cooperation with the VKS.

We demonstrated that development of clinical pathways for IEMs is feasible and we were able to reach national consensus. At this time, Dutch pathways are publically available for 20 diseases including urea cycle defects, organic acidurias, mitochondrial fatty acid oxidation disorders, galactosemia, phenylketonuria, tyrosinemia, glycogen storage disorders, congenital disorder of glycosylation type 1a, and Niemann Pick type c [21].

## Abbreviations

VKS: Dutch society for children and adults with an inborn error of metabolism; IEMs: Inborn errors of metabolism; PKU: Phenylketonuria.

## Competing interests

None of the authors, or any of the members in the working group, have financial or non-financial competing interests to declare.

## Authors’ contributions

SD has made substantial contributions to conception and design, has been involved in drafting of the manuscript and has given final approval of the version to be published. INK has made substantial contributions to conception and design, has been involved in drafting of the manuscript and has given final approval of the version to be published. MJK has made substantial contributions to conception and design, has been involved in drafting of the manuscript and has given final approval of the version to be published. CEMH has been involved in drafting of the manuscript and has given final approval of the version to be published. HM has made substantial contributions to conception and design, has been involved in drafting of the manuscript and has given final approval of the version to be published. AK has been involved in drafting of the manuscript and has given final approval of the version to be published. MR has been involved in drafting of the manuscript and has given final approval of the version to be published. FJS has been involved in drafting of the manuscript and has given final approval of the version to be published. AMB has made substantial contributions to conception and design, has been involved in drafting of the manuscript and has given final approval of the version to be published. All members of the *The Dutch working Group on clinical pathways for inborn errors of metabolism* have given final approval of the version to be published.

## Authors’ information

Dutch working Group on clinical pathways for inborn errors of metabolism.

Folkert. W. Asselbergs^1^, Christiaan Blank^2^, Terry G.J. Derks ^8^, Eugène F. Diekman^2^, Monique E. Dijsselhof^6^, Marc Engelen^7^, Peter M. van Hasselt^2^, Nienke M. ter Horst^6^, Dorine A.M. van den Hurk^3^, Mirian C.H. Janssen^9^, Francois P.J. Karstens^11^, Elles van der Louw^12^, Eva Morava^10^, Joost Nicolai ^14^, Ludo van de Pol^2^, Bwee Tien Poll-The ^5^, Estela Rubio-Gozalbo^15^, G. Peter A. Smit^8^, Jessica de Ruijter^5^, Corrie Timmer^3^, Catharina M.L. Touw ^8^, Gepke Visser^2^, Harold W. de Valk^4^, Frits A. Wijburg^5^, Monique Williams^13^.

Departments of Cardiology^1^, Pediatrics^2^, Dietetics^3^ and Internal Medicine ^4^, University Medical Center, Utrecht, The Netherlands.

Department of Pediatrics^5^, Dietetics^6^, Neurology^7^, Emma Children’s Hospital, Academic Medical Center, Amsterdam, The Netherlands.

Section of metabolic diseases^8^, Beatrix Children’s Hospital, University Medical Center Groningen, University of Groningen, Groningen, The Netherlands.

Department of Internal Medicine^9^ and Pediatrics^10^, Unitversity Medical Center st Radboud, Nijmegen.

Department of Internal Medicine^11^, Dietetics^12^ and Pediatrics^13^, Erasmus MC, Rotterdam, The Netherlands.

Department of Neurology^14^ and Pediatrics^15^, University Hospital Maastricht, Maastricht, The Netherlands.
